# Supplementary material for: Development of chemokine network inhibitors using combinatorial saturation mutagenesis
Source: Commun Biol. 2025 Apr 3;8:549. doi: 10.1038/s42003-025-07778-6 (PMC11969024; doi:10.1038/s42003-025-07778-6)
Supplement: Supplementary file 4 — Supplementary Data 2 [file 42003_2025_7778_MOESM4_ESM.zip › SupplementaryData3/scripts/xgeo.tar!/./script/analysis/JK/GSE226888_homo_stimulated_islet_boxplot.pdf]

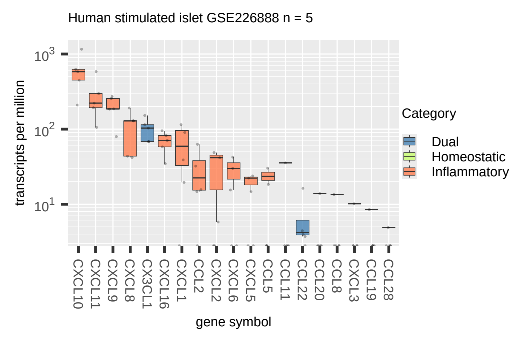

Human stimulated islet GSE226888 n = 5
transcripts per million
10^3
10^2
10^1
CXCL10
CXCL11
CXCL9
CXCL8
CX3CL1
CXCL16
CXCL1
CCL2
CXCL2
CXCL6
CXCL5
CCL5
CCL11
CCL22
CCL20
CCL8
CXCL3
CCL19
CCL28
gene symbol
Category
Dual
Homeostatic
Inflammatory
